# Identifying structures of continuously-varying weighted networks

**DOI:** 10.1038/srep26649

**Published:** 2016-05-31

**Authors:** Guofeng Mei, Xiaoqun Wu, Guanrong Chen, Jun-an Lu

**Affiliations:** 1 School of Mathematics and Statistics, Wuhan University, Hubei 430072, China; 2 Computational Science Hubei Key Laboratory, Wuhan University, Wuhan 430072, China; 3 Department of Computer Science, University of California, Davis CA 95616, USA; 4 Department of Electronic Engineering, City University of Hong Kong, Hong Kong, China

## Abstract

Identifying network structures from dynamical observations is a fundamental problem currently pervading scientific research on complex systems, as understanding and modeling the structure of a complex network will lead to greater knowledge of its evolutionary mechanisms and to a better understanding of its functional behaviors. Usually, one needs to identify a network’s structure through a limited number of observations. Particularly, couplings of many real-world networks are sparse and continuously varying with time. In this study, a new framework is developed via optimization for identifying structures of continuously-varying weighted networks formed by sparsely-connected dynamical systems. Furthermore, a regularization technique is employed to increase the numerical stability of the parameter estimation algorithm. Three numerical examples are provided to illustrate the feasibility and effectiveness of the proposed identification method. In comparison with other existing techniques, the main advantages of our method include its ability to identify structures of continuously-varying weighted networks in addition to static ones, as well as its requirement of a relatively small number of observations. The proposed method has a potential applicability to a variety of evolving complex dynamical networks.

Time-varying networks describe a large number of systems whose constituents and interactions evolve over time^1^,^2^,^3^. This subject has attracted extensive attention from researchers in various fields. For instance, Starnini *et al.* proposed several immunization strategies for epidemic processes in time-varying networks^2^, Amritkar *et al.* studied the synchronization properties of coupled dynamics on time-varying networks and the corresponding time-average network^4^, and Nicola *et al.* explored the random walk process in a fairly general class of time-varying networks^5^. Indeed, complex systems taking the form of time-varying networks are ubiquitous. Some plausible representations of the relational information among entities in dynamical systems are time-varying networks that are topologically rewiring and semantically evolving over time^6^,^7^,^8^, such as a social community, living cells, e-mail messages, mobile telephone calls, functional brain networks, food webs and other networks of species. It is noteworthy that topological structures of complex networks play an important role in determining their evolutionary dynamics and functional behaviors, and may have significant consequences for many real-world applications^9^. However, in many practical situations, the precise structure of a complex dynamical network is unknown or uncertain. Therefore, to find a general solution to the structure identification problem of complex networks is of primary importance.

Many achievements have been made on structure identification of complex networks^10^,^11^,^12^,^13^,^14^,^15^,^16^. For example, Zhang *et al.* constructed an auxiliary complex network in a very general form and designed some adaptive controllers to identify network structures based upon generalized outer synchronization^10^, Wu *et al.* used Granger causality test to infer network structures^11^,^12^, Wang *et al.* estimated network parameters based on compressive sensing^13^,^14^, Kolar *et al.* presented two new machine learning methods for estimating time-varying networks based on a temporally smoothed *l*_1_-regularized logistic regression formalism^15^, and Rao *et al.* used a state-space model to infer time-varying network topologies from gene expression data^16^. However, most if not all of these studies focus on the case that network structures are static, and those works regarding estimating time-varying structures of networks only care about topological changes in the structures, not about continuous changes of coupling weights. Yet, networks are not only specified by their structures but also by the dynamics of information taking place on the structure^17^. Examples include a social network where there exists stronger or weaker social ties between individuals, a metabolic network where there are more or less flux along particular reaction pathways, a food web where there are varying energy or carbon flow between predator-prey pairs^18^, among many others. Thus it is practical to assign varying weights for each edge of a complex network without changing the structure^17^.

In this paper, we propose a method to identify structures of networks whose edge weights continuously vary with time. In many situations, the interactions among elements of a large system are rapidly changing and are usually characterized by processes whose timing and duration are defined on a very short temporal scale^3^. Since interactive weights among elements in a real-world network sometimes are varying continuously with time, the assumption of static structures or activity-driven varying structures is sometimes inappropriate. Continuously-varying structures should thus be taken into account for analysis of this kind of realistic complex networks. Furthermore, due to the fact that the structure of a real-world network normally is sparse and there is only a limited number of observations, we propose an optimization framework to identify the continuously-varying weights of a complex network. In designing the algorithm, a regularization technique is incorporated, which led to an efficient and robust scheme as finally verified by accurate or noisy data sets observed from a 6-node directed chaotic network, a 50-node undirected network, as well as a 50000-node small-world network.

## Network analysis

### Mathematical notations

Some necessary notations that will be used throughout the paper are introduced. **x**^⊤^ (or *M*^⊤^) denotes the transpose of a vector **x** (or a matrix *M*), 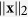 is the Euclidean-norm of **x**, 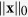 is the *l*^0^ norm of **x**, ⊗ represents the Kronecker product, 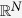 is the *N*-dimensional real space, 
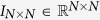
 denotes the identity matrix of order *N*.

Consider the following complex dynamical network consisting of *N* identical nodes with continuously-varying couplings:


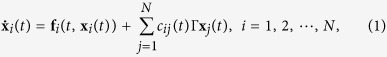


where 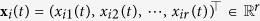 is the state vector of the *i*-th node, and 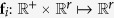 is a smooth nonlinear vector-valued function governing the dynamics of the *i*-th node. Matrix 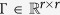 is the inner coupling matrix determining the interaction of component variables. The configuration matrix 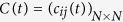 describes the continuously-varying topology of the weighted network. Specifically, at time point *t*, if there is a link from node *j* to node *i* (*j* ≠ *i*), then the coupling strength *c*_*ij*_(*t*) > 0, otherwise *c*_*ij*_(*t*) = 0. The diagonal entries of matrix *C*(*t*) are defined as


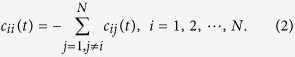


Denote 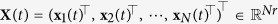 and 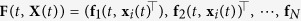

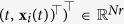
. Then, network (1) can be rewritten as


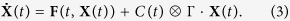


Furthermore, by denoting 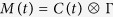 and 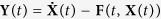, one has


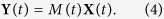


In general, 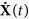 can be approximated by its first-order difference quotient, that is,


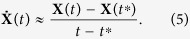


Here, t* is a time point that is sufficiently close to *t* so that 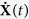 can be more accurately approximated.

Let 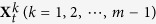 represent the observed node dynamics **X**(*t*) at time point *t* for the *k*-th time, where *m* − 1(*m* ∈ 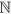) is the total number of observations. Then one has


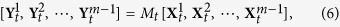


where *M*_*t*_ = *M*(*t*) and 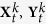 represent the *k*-th observations of **X**(*t*) and **Y**(*t*), respectively.

From Eq. (2), one has 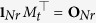, where 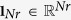 is a row vector whose elements are ones and 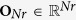 is a row vector whose elements are zeros. Let 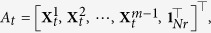 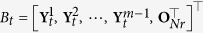. Then, one has


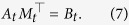


If every row of *C*(*t*) ⊗ Γ is a sparse vector, the above problem can be translated into solving the following minimization problem:


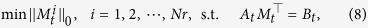


where index *i* represents the *i*-th row of matrix *M*_*t*_. To ensure the restricted isometry property, one will normalize *A*_*t*_ with dividing elements in each column with the Euclidean norm of that column^13^.

## Results

In this section, three numerical examples are provided to illustrate the effectiveness of the identification technique proposed in the preceding section. In the first two examples, each node in the network represents a well-known three-dimensional chaotic Lorenz system, as described by


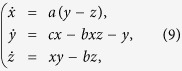


where *a* = 10, *b* = 8/3, *c* = 28.

In the following numerical simulations, the fourth-order Runge–Kutta method is employed to solve the ordinary differential equations of the dynamical network. The *m* − 1 observations are collected by solving the ordinary differential equations in Eq. (3) with *m* − 1 sets of different initial values.

### Numerical validation on a 6-node weighted directed network

Consider a weighted directed network (1) consisting of 6 Lorenz oscillators, with the continuously-varying structure matrix being


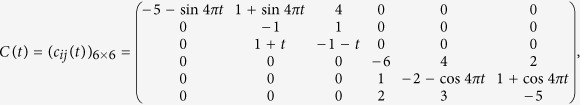


and the inner coupling matrix being Γ = *I*_3×3_. In the following simulations, it is always assumed that the observed **X**(*t*) in Eq. (4) is accurate. The time step used in the Runge-Kutta method for generating data is *h* = 10^−6^, and 11(*m* = 12) different trajectories are generated from (1) starting from 11 sets of random initial values. Therefore, at time point *t*, 11 observations can be collected to identify instantaneous network structures.

**Figure 1:**
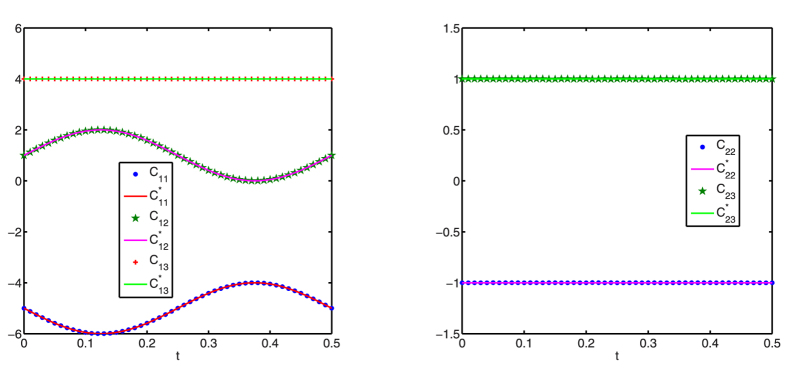
Structure identification of the 6-node network with accurate observations. Left: *c*_1*j*_(*t*) (*j* = 1, 2, 3); right: *c*_2*j*_(*t*) (*j* = 2, 3).

**Figure 2:**
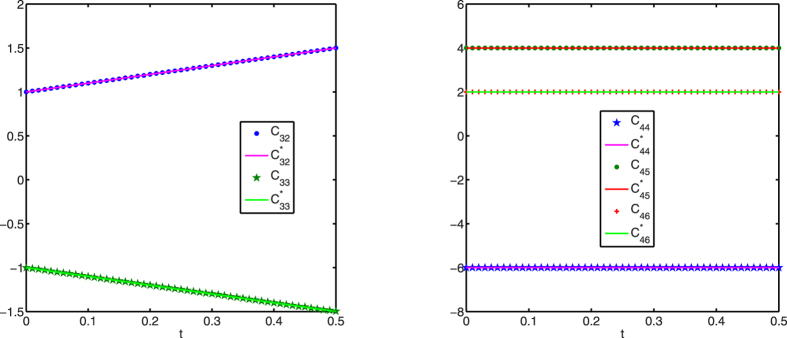
Structure identification of the 6-node network with accurate observations. Left: *c*_3*j*_(*t*) (*j* = 2, 3); right: *c*_4*j*_(*t*) (*j* = 4, 5, 6).

**Figure 3:**
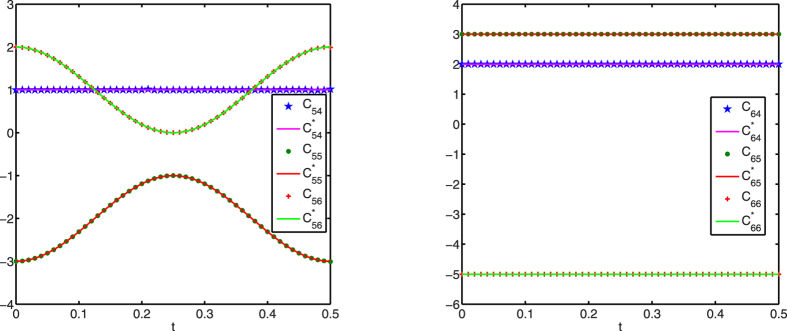
Structure identification of the 6-node network with accurate observations. Left: *c*_5*j*_(*t*) (*j* = 4, 5, 6); right: *c*_6*j*_(*t*) (*j* = 4, 5, 6).

Figures 1, 2, 3 display successful identification of *C*(*t*) from noise-free dynamical observations, where only nonzero elements of matrix *C*(*t*) are displayed. In all the figures, the dots (*c*_*ij*_) represent the estimated values at different time points and the lines 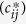 are the original curves of *c*_*ij*_(*t*). From these figures, it is obvious that the estimated values match perfectly with the original structures.

**Figure 4:**
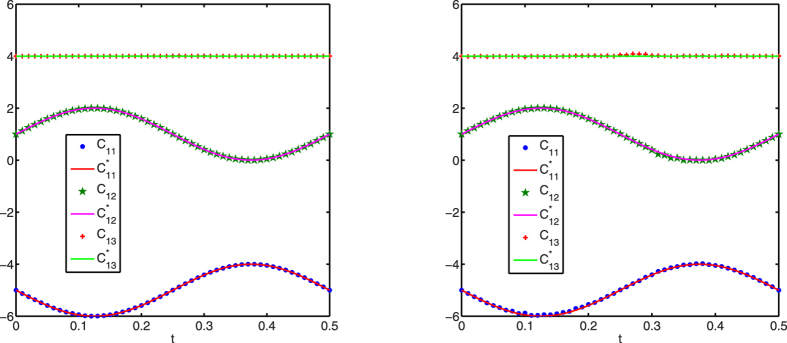
Structure identification of the 6-node network with noisy observations. Left: noise satisfies a uniform distribution on the interval [0, 0.01]; right: noise satisfies a uniform distribution on the interval [0, 0.1].

In real-world applications, observation noise commonly exists. Thus, in order to test the robustness of the proposed optimization-based structure identification algorithm in practical scenarios, two more numerical simulations are carried out. Figure 4 presents identification results from noisy observations, where observed node dynamics are assumed to be contaminated by white noise. The left panel of Fig. 4 shows the result when observation noise satisfying a uniform distribution on the interval [0, 0.01] is inserted. It is seen that the varying structures are still correctly identified. In the right panel, the noise is stronger and satisfies a uniform distribution over the interval [0, 0.1]. The inferred values still match those of the original ones, though there is some slight disagreement caused by noise. This example illustrates the strong robustness of the proposed approach.

**Figure 5:**
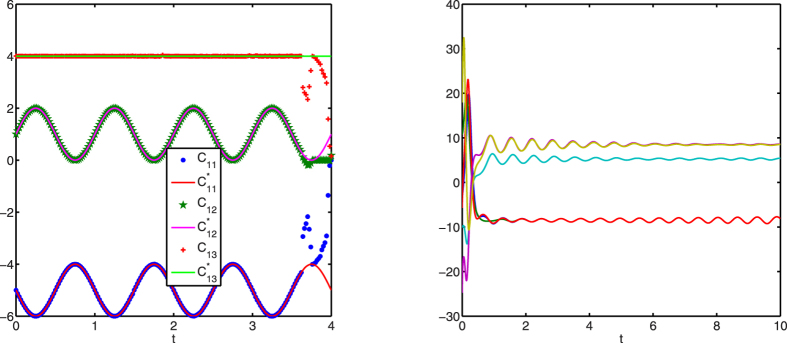
Time-varying structures can be successfully identified from transient non-synchronous network dynamics. Left: identification of *c*_1*j*_(*t*) (*j* = 1, 2, 3); right: time evolution of the first component variable of each node in one trajectory.

As is known, synchronization is an obstacle to topology identification^19^,^20^. Figure 5 displays the identification results with a longer time span without noise perturbation. From the left panel, it is observed that structures can be correctly identified during the time period [0, 3.6). At about *t* = 3.6, the structures cannot be identified anymore. It is because that the network gets into partial synchronization, which results in identification failure. The right panel of Fig. 5 shows the time evolutions of the first component variables of all the nodes starting from one set of initial values, which arrive at synchronization after a short transient period. Therefore, it is important to ensure that nodes in a dynamical network are not in any kind of synchronization status, otherwise structure identification is likely to fail.

### Numerical validation on a 50-node small-world network

**Figure 6:**
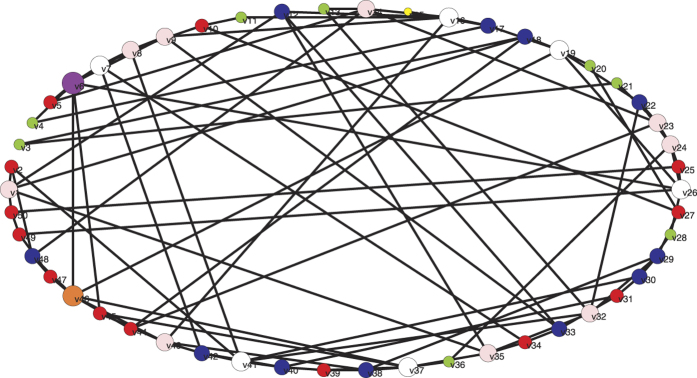
A W–S small-world network generated with rewiring probability *p* = 0.5.

In this subsection, the proposed method is tested on a network consisting of 50 nodes. The Watts–Strogatz (W–S) algorithm^21^ is employed here to generate a small-world network. Specifically, start with a ring of *N* = 50 nodes, each connecting to its *k* = 4 nearest neighbours by undirected edges. Then, rewire each edge in such a way that the beginning end of the edge is kept but the other end is disconnected with probability *p* and then reconnected to another node randomly selected from the network. A W–S small-world network generated with rewiring probability *p* = 0.5, as shown in Fig. 6, is employed here for illustration. For simplicity, assume *c*_13_(*t*) = *c*_31_(*t*) = |sin *t*|, *c*_24_(*t*) = *c*_42_(*t*) = ln(1 + *t*). For all the other couplings, if there is a link from node *j* to node *i* (*j* ≠ *i*) at time point *t*, then *c*_*ij*_(*t*) = 1, otherwise *c*_*ij*_(*t*) = 0. The diagonal entries of matrix *C*(*t*) satisfy 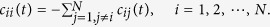 The inner coupling matrix is Γ = *I*_3×3_.

In the following simulations, it is always assumed that node dynamics can be exactly observed. The time step used in the Runge-Kutta method for generating data is still *h* = 10^−6^, and *m* − 1(*m* = 101) different trajectories are generated from (1) starting from *m* − 1 sets of different initial values.

**Figure 7:**
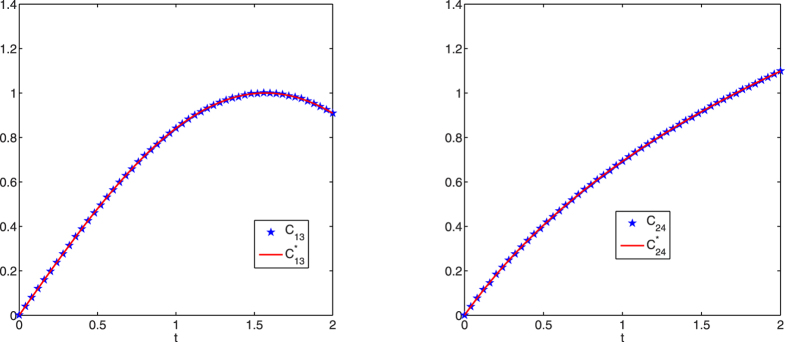
Structure identification of the 50-node small-world network with accurate observations. Left: identification of *c*_13_(*t*); right: identification of *c*_24_(*t*).

For brevity, Fig. 7 only shows the identification results for continuously-varying couplings *c*_13_(*t*) and *c*_24_(*t*). It is obvious that the couplings are correctly identified at different time points, which again illustrates the effectiveness of the proposed identification method from limited observations.

### Numerical validation on a 50000-node small-world network

In this example, the one-dimensional multi-agent system proposed by Saber *et al.*^22^ is taken as node dynamics. Denote *x*_*i*_ as the state of agent *i* and suppose that the dynamics of agent *i* is described by


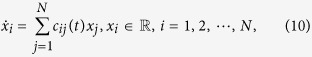


where *N* is the number of agents. The collective dynamics of the group of interacting agents can be rewritten as


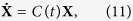


where 
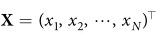
.

An undirected small-world network consisting of 50000 agents is used to illustrate the effectiveness of the proposed method. The network starts with a ring of *N* = 50000 nodes, each connects to its *k* = 8 nearest neighbours by undirected edges that are rewired with probability *p* = 0.4. Specifically, assume 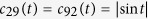, 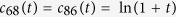. For all the other couplings, if there is a link between node *i* and node *j* (*j* ≠ *i*) at time point *t*, then *c*_*ij*_(*t*) = *c*_*ji*_(*t*) = 1, otherwise *c*_*ij*_(*t*) = 0. The diagonal entries of matrix *C*(*t*) satisfy 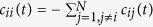 for 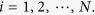


The time step used in the Runge-Kutta method for generating data is *h* = 10^−6^, and *m* − 1(*m* = 10000) different trajectories are generated from (1) starting from *m* − 1 sets of different initial values.

**Figure 8:**
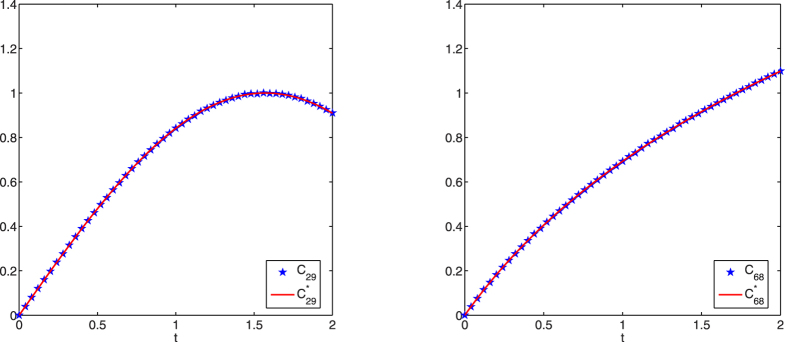
Structure identification of the 50000-node small-world network with accurate observations. Left: identification of *c*_29_(*t*); right: identification of *c*_68_(*t*).

For brevity, Fig. 8 only shows the identification results for continuously-varying couplings *c*_29_(*t*) and *c*_68_(*t*). It is obvious that the couplings are correctly identified at different time points, which again illustrates the effectiveness of the proposed identification method from limited observations.

## Discussion

Identifying network structures is not only a very important research topic itself but also has applications in a variety of domains. Various methods had been presented to solve the problem of inferring structures of static networks from observations and perhaps through synchronization or control of dynamics. Within the growing body of work concerning time-varying networks, few studies have focused on the issue of identifying structures of continuously-varying weighted networks. In this paper, we have proposed a new method based on optimization to identify structures of complex dynamical networks with continuously-varying weights from limited observations. Two problems, which can be used to solve optimal problems and increase the numerical stability, have been established by rigorous mathematical analysis. In comparison with other existing methods, the main advantages of this approach are that it can handle the challenging identification problem of structures of continuously-varying weighted networks other than static ones. In addition, it only requires a relatively small number data, so it can dramatically reduce the difficulty in obtaining large numbers of observations. Furthermore, by taking into consideration observation noise, the robustness of the method has been demonstrated. In the proposed algorithm, a regularization method has been incorporated, which can avoid large deviations caused by small singular values thereby further increase the numerical stability. Therefore, the method provides a sensible way to identify structures of continuously-varying weighted networks from observations, which has a clear potential applicability to a wide variety of complex dynamical networks.

## Methods

### Theoretical analysis and algorithm design (more details can be found in the SI)

Here, we design an algorithm to solve optimization problem (8). Topology identification for a complex network with limited observations can be casted into reconstructing a vector 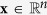 from an underdetermined system of linear equations which has more unknowns than equations. The problem can be described by 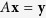, where *A* is an *m* × *n* matrix, 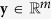, and *m* is the number of measurements, with *m* < *n*. Thus, the underdetermined system *A***x** = **y** may have infinitely many solutions. In order to find a solution to such a system, preferably optimal in some sense, one must impose extra constraints as appropriate. In this paper, it is assumed that matrix *A* has a full row-rank and **x** is sparse. In this case, one can minimize the number of nonzero components of **x** to obtain the sparsest solution to *A***x** = **y**, that is, to solve the following optimization problem^23^:


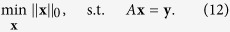


where 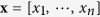. The minimization problem (12) can be transformed to the following problem^24^,^25^:


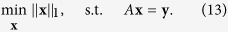


where 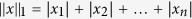 is the *l*^1^ norm of the sparse vector *x*. However, when the matrix *A* is ill-conditioned and **y** cannot be accurately observed, numerical instability will arise (more details can be found in the SI). In order to increase the numerical stability, a classical regularization is introduced here. The regularized problem is


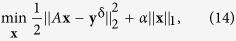


where *α* = *α*(*δ*) > 0 is the regularization parameter used to avoid large deviation from the optimal solution. Here, 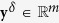 is an erroneous observation, *δ* is the noise level and 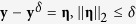, **η** represents the observation error.

Therefore, the following problem is formulated for the case with accurate observation data.

**Problem 0.1** By introducing multipliers, we can reformulate the augmented Lagrangian of problem (13) as


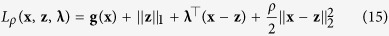


where 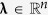 and *ρ* > 0 are the Lagrange multipliers and penalty parameter, respectively; **z** is an auxiliary vector that has the same dimension as **x** and satisfies the constraint **x** − **z** = 0; **g**(**x**) is the indicator function of 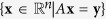 to ensure **x** satisfying the constraint *A***x** = **y**.

For noisy observations, one has the following reformulation.


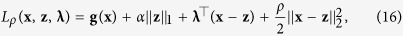


**Problem 0.2** *When noise exists in observations, the optimal solution to problem (13) can be approximated by the solution to the optimization problem (14). By introducing multipliers, one has*

where 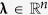 and *ρ* > 0 are the Lagrange multipliers and penalty parameter, respectively; **z** is an auxiliary vector that has the same dimension as **x** and satisfies the constraint **x** − **z** = 0; 
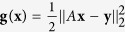
.

**Remark 0.1** *Solving Problems 0.1 and 0.2, one can obtain solutions to the convex optimization problems (13) and (14) using the well-known ADMM algorithm*^26^.

## Additional Information

**How to cite this article**: Mei, G. *et al.* Identifying structures of continuously-varying weighted networks. *Sci. Rep.* **6**, 26649; doi: 10.1038/srep26649 (2016).

## Supplementary Material

Supplementary Information
