# Supplementary material for: Identifying structures of continuously-varying weighted networks
Source: Sci Rep. 2016 May 31;6:26649. doi: 10.1038/srep26649 (PMC4901193; doi:10.1038/srep26649)
Supplement: Supplementary Information [file srep26649-s1.pdf]

Supplementary Information Identifying structures of continuously-varying weighted networks

Guofeng Mei, Xiaoqun Wu, Guanrong Chen, and Jun-an Lu

Theoretical analysis and algorithm design

Topology identification for a complex network with limited observations can be casted into reconstructing a vector $\mathbf{x} \in \mathbb{R}^n$ from an underdetermined system of linear equations which has more unknowns than equations. The problem can be described by $A\mathbf{x} = \mathbf{y}$, where A is an $m \times n$ matrix, $\mathbf{y} \in \mathbb{R}^m$, and m is the number of measurements, with $m < n$. Thus, the underdetermined system $A\mathbf{x} = \mathbf{y}$ may have infinitely many solutions. In order to find a solution to such a system, preferably optimal in some sense, one must impose extra constraints as appropriate. In this paper, it is assumed that matrix A has a full row-rank and $\mathbf{x}$ is sparse. In this case, one can minimize the number of nonzero components of $\mathbf{x}$ to obtain the sparsest solution to $A\mathbf{x} = \mathbf{y}$, that is, to solve the following optimization problem [1]:

$$\min_{\mathbf{x}} \|\mathbf{x}\|_0, \quad \text{s.t.} \quad A\mathbf{x} = \mathbf{y}. \tag{1}$$

The l^0 norm of $\mathbf{x} = [x_1, \cdots, x_n]^\top$ is defined as the number of non-zero entries of $\mathbf{x}$. In other words, if one defines [2]

$$v(x) = \begin{cases} 1, & x \neq 0, \\ 0, & x = 0, \end{cases} \tag{2}$$

then

$$\|\mathbf{x}\|_0 = \sum_{i=1}^{n} v(x_i). \tag{3}$$

It is clear that the discontinuity of the l^0 norm of a vector $\mathbf{x}$ is caused by the discontinuity of the function v. However, the minimization problem (1) can be transformed to the following problem [3], [4]:

$$\min_{\mathbf{x}} \|\mathbf{x}\|_1, \quad \text{s.t. } A\mathbf{x} = \mathbf{y}. \tag{4}$$

where $\|\mathbf{x}\|_1 = |x_1| + |x_2| + ... + |x_n|$ is the l^1 norm of the sparse vector $\mathbf{x}$. When the matrix A is ill-conditioned and $\mathbf{y}$ cannot be accurately observed, numerical instability will arise. To illustrate it, let $\mathbf{y} = A\mathbf{x}_{true}$, $\mathbf{y} - \mathbf{y}^\delta = \boldsymbol{\eta}$, $\|\boldsymbol{\eta}\|_2 \leq \delta$. Here, $\mathbf{x}_{true}$ represents the true or accurate source, $\mathbf{y}^\delta$ is an erroneous observation, δ is the noise level and $\boldsymbol{\eta}$ represents the observation error. A has a singular value decomposition (SVD) of the form

$$A = UDV^\top, \tag{5}$$

where $U = [\mathbf{u}_1, \mathbf{u}_2, \cdots, \mathbf{u}_m]$ and $V = [\mathbf{v}_1, \mathbf{v}_2, \cdots, \mathbf{v}_n]$ are matrices of order m and n, respectively, with column vectors $\mathbf{u}_i$ and $\mathbf{v}_i$ being the right and left singular vectors of A, $D = [\text{diag}(s_1, s_2, \cdots, s_m), \mathbf{O}_{m\times n-m}]$ is an $m \times n$ rectangular diagonal matrix with non-negative real numbers on the diagonal, and $s_i \ (i = 1, 2, \cdots, m, \ s_1 < s_2 \cdots < s_m)$ are the singular values of A. Furthermore, one has

$$A^\dagger \mathbf{y}^\delta = VD^\dagger U^\top \mathbf{y}^\delta = \mathbf{x}_{true} + \sum_{i=1}^{m} s_i^{-1}(\boldsymbol{u}_i^\top \boldsymbol{\eta})\mathbf{v}_i, \tag{6}$$

where $A^\dagger$ and $D^\dagger$ are respectively the Moore–Penrose pseudo-inverse [5] of A and D. Thus, one obtains from Eq. (6) that

$$\|A^\dagger \mathbf{y}^\delta - A^\dagger \mathbf{y}\|_2 \to \infty \text{ when } s_i \to 0. \tag{7}$$

Therefore, when matrix A is ill-conditioned and $\mathbf{y}$ is not accurately observed, numerical instability will arise in solving $A\mathbf{x} = \mathbf{y}$. In order to increase the numerical stability, a classical Tikhonov regularization is introduced here. The regularized problem is

$$\min_{\mathbf{x}} \|A\mathbf{x} - \mathbf{y}^\delta\|_2^2 + \alpha\|\mathbf{x}\|_1, \tag{8}$$

where $\alpha = \alpha(\delta) > 0$ is the regularization parameter used to avoid large deviation from the optimal solution.

The authors acknowledge support from the National Natural Science Foundation of China under Grant Nos. 61573263, 61174028 and 11172215, and the Hong Kong Research Grants Council under the GRF Grant CityU-11208515.

G. Mei, X. Wu, and J. Lu are with the School of Mathematics and Statistics, Wuhan University, Hubei 430072, China, X. Wu is also with Computational Science Hubei Key Laboratory, Wuhan University, Wuhan 430072, China and Department of Computer Science, University of California, Davis CA 95616, USA (Email: xqwu@whu.edu.cn).

G. Chen, Department of Electronic Engineering, City University of Hong Kong, Hong Kong, China.

References

[1] D. L. Donoho, "Compressed sensing," *IEEE Transactions on Information Theory*, vol. 52, no. 4, pp. 1289–1306, 2006.

[2] G. H. Mohimani, M. Babaie-Zadeh, and C. Jutten, "Fast sparse representation based on smoothed l_0 norm," in *Independent Component Analysis and Signal Separation*, pp. 389–396, Springer, 2007.

[3] E. J. Candes and T. Tao, " Decoding by linear programming," *IEEE Transactions on Information Theory*, vol. 51, no. 12, pp. 4203–4215, 2006.

[4] D. L. Donoho, " For most large underdetermined systems of linear equations the minimal l^1-norm solution is also the sparsest solution," *Communications on Pure and Applied Mathematics*, vol. 59, no. 6, pp. 797–829, 2006.

[5] H. W. Engl, M. Hanke, and A. Neubauer, *Regularization of inverse problems*, vol. 375. Springer Science and Business Media, 1996.
